# Octadecyl 3-(3, 5-di-tert-butyl-4-hydroxyphenyl) propanoate nanosponges: enhanced antibacterial and antibiofilm activity against multidrug-resistant *Klebsiella pneumoniae* with synergistic ceftriaxone combination

**DOI:** 10.3389/fcimb.2026.1867536

**Published:** 2026-07-16

**Authors:** Sayed E. El-Sayed, Khaled M. Aboshanab, Hesham Ali El Enshasy, Mohamed H Al-Agamy, Khalid Alyahya, Ann A. Elshamy

**Affiliations:** 1 Department of Microbiology and Immunology, Faculty of Pharmacy, Ahram Canadian University, Sixth of October City, Giza, Egypt; 2 Department of Microbiology and Immunology, Faculty of Pharmacy, Ain Shams University, Cairo, Egypt; 3 Innovation Centre in Agritechnology for Advanced Bioprocessing (ICA), Universiti Teknologi Malaysia (UTM), Pagoh, Johor, Malaysia; 4 Department of Pharmaceutics, College of Pharmacy, King Saud University, Riyadh, Saudi Arabia

**Keywords:** antibiofilm activity, antimicrobial synergy, ceftriaxone, *Klebsiella pneumoniae*, multidrug resistance, nanosponge, ODHP

## Abstract

Global public health is seriously threatened by the rising incidence of multidrug-resistant (MDR) *Klebsiella pneumoniae*, underscoring the critical need for innovative antimicrobial approaches. Herein, the antibacterial and antibiofilm activities of octadecyl 3-(3,5-di-tert-butyl-4-hydroxyphenyl) propanoate (ODHP) and its nanosponge formulation (ODHP-NS) were evaluated against clinical MDR *K. pneumoniae* isolates, and the potential synergistic interaction between ODHP-NS and ceftriaxone was investigated using the checkerboard assay and further confirmed by time–kill kinetics. Results showed that ODHP-NS exhibited enhanced antibacterial activity compared to ODHP, with lower MIC_50_ and MIC_90_ values (32 vs. 96 µg/mL and 128 vs. 256 µg/mL, respectively), indicating improved efficacy following nanosponge formulation. Both ODHP and ODHP-NS demonstrated significant inhibition of biofilm formation at sub-inhibitory concentrations (1/2 and 1/4 MIC), with ODHP-NS showing significantly higher antibiofilm activity (*p* < 0.0001). Fractional inhibitory concentration index (FICI) values indicated synergistic to additive effects in most tested combinations, and the time–kill kinetics demonstrated a pronounced bactericidal effect, achieving a ≥3 log_10_ reduction in bacterial counts compared to the most active single agent, with no evidence of bacterial regrowth over 24 h. It was concluded that nanosponge formulation significantly enhanced the antibacterial and antibiofilm properties of ODHP and effectively potentiated the activity of ceftriaxone against MDR *K. pneumoniae*. This study highlights ODHP-NS as a potential antimicrobial adjuvant for combating MDR *K. pneumoniae* infections.

## 1 Introduction

The global increase in the rates of antimicrobial resistance (AMR) has transformed a wide range of bacterial infections into life-threatening conditions, where multidrug-resistant (MDR) *Klebsiella pneumoniae*, classified as an ESKAPE organism (Boucher et al., 2009), is one of the most challenging pathogens in clinical settings (Boucher et al., 2009; Chen et al., 2024). This bacterium is a major cause of several potentially fatal nosocomial infections as well as illnesses linked to healthcare (Asokan et al., 2025; Boucher et al., 2009; Mukherjee et al., 2021; Paczosa and Mecsas, 2016). The World Health Organization (WHO) has listed *K. pneumoniae* as a critical-priority pathogen requiring new antimicrobial strategies (WHO, 2024). The clinical management of MDR *K. pneumoniae* is highly compromised by its ability to acquire resistance genes (Navon-Venezia et al., 2017). The antimicrobial resistance of *K. pneumoniae* is further enhanced through its biofilm formation ability, making it resilient in hostile environments. The combination of multidrug resistance and biofilm formation creates a dual therapeutic challenge that conventional antibiotics alone cannot adequately address, leading to treatment failures (Roustaye Gourabi et al., 2026). The currently limited availability of effective antimicrobials has resulted in emerging research concerned with novel therapeutic options such as antibiotic combinations, phage therapy, antimicrobial peptides, natural products or their bioactive components, and nanoparticles (Li et al., 2024).

Among natural products, phenolic compounds and their derivatives have demonstrated antibacterial and antibiofilm activities against MDR pathogens (Borges et al., 2016). Octadecyl 3-(3,5-di-tert-butyl-4-hydroxyphenyl) propanoate (ODHP) is a long-chain phenolic ester identified by El-Sayed et al. as an antifungal metabolite produced by *Alcaligenes faecalis* strain MT332429. While its antifungal properties have been characterized, the antibacterial activity of ODHP against MDR *K. pneumoniae* was not evaluated (El-Sayed et al., 2020). Nanotechnology-based drug delivery systems, particularly nanosponges, offer a solution to the poor aqueous solubility that limits the bioavailability of many hydrophobic natural products. Nanosponges are cross-linked polymeric structures with a three-dimensional porous network that enables high entrapment efficacy for both hydrophilic and hydrophobic drugs. Due to their porous structure, nanosponges can encapsulate molecules within their cavities, protect them from degradation, and allow their controlled and sustained release over extended periods (Sherje et al., 2017). Cyclodextrin-based nanosponges have been successfully used as nanocarriers for antimicrobial agents, demonstrating improved stability and prolonged antimicrobial activity against *Pseudomonas aeruginosa* and *Staphylococcus aureus*, which are among the pathogens that often cause hospital-acquired infections (Zagami et al., 2023). El-Sayed et al. have recently developed an ODHP-loaded nanosponge formulation (ODHP-NS) and characterized its physicochemical properties; however, its antibacterial and antibiofilm efficacy against MDR *K. pneumoniae* was not explored (El-Sayed et al., 2024).

Combination therapy is a strategy employed to combat MDR pathogens and restore the activity of conventional antibiotics (Tyers and Wright, 2019). Recent studies have shown that the 3^rd^ generation cephalosporin “ceftriaxone” can exhibit synergistic effects when combined with appropriate adjuvants such as β-lactamase inhibitor BLI-489. This combination demonstrated significant synergistic activity against NDM-producing *K. pneumoniae*, with a fractional inhibitory concentration index (FICI) of 0.25, indicating strong synergy. This combination also inhibited biofilm formation and showed reduced hemolytic activity compared to each compound alone, suggesting higher biocompatibility (Damaceno et al., 2026). The potential for ODHP-NS to synergize with ceftriaxone against MDR isolates has not been investigated. Given that ceftriaxone is widely used but increasingly ineffective against MDR *K. pneumoniae* due to widespread Extended-spectrum β-lactamase (ESBL) and carbapenemase production, identifying an adjuvant that restores its activity would have significant clinical value. In this study, we aimed to evaluate and compare the antibacterial activity of free ODHP versus ODHP-NS against MDR *K. pneumoniae* isolates, to assess the antibiofilm activity of both agents at sub-inhibitory concentrations, to investigate the synergistic potential of ODHP-NS in combination with ceftriaxone using checkerboard and time–kill kinetic assays, and to determine whether nanosponge formulation significantly enhances the therapeutic potential of ODHP. We hypothesized that nanosponge encapsulation of ODHP would enhance its aqueous solubility, thereby improving its antibacterial and antibiofilm activity against MDR *K. pneumoniae* compared to the free compound. Furthermore, we hypothesized that ODHP-NS would synergize with ceftriaxone, thereby restoring ceftriaxone efficacy against resistant isolates.

## 2 Materials and methods

### 2.1 Bacterial isolates and ethical statement

A total of 28 clinical isolates of *K. pneumoniae* were used in this study; they were obtained from the archived culture collection of the Microbiology and Immunology Department, Faculty of Pharmacy, Ain Shams University, Cairo, Egypt. These isolates were originally recovered from clinical specimens in three previous studies (Elshamy et al., 2018, 2020, 2021). The *K. pneumoniae* isolates have been identified using 16S ribosomal RNA sequencing and confirmed using the VITEK^®^ 2 Compact automated platform (bioMérieux, France) for both biochemical and antibiotic resistance analysis.

All isolates had been stored at -80 °C in Luria-Bertani (LB) broth supplemented with 25% glycerol since their original collection and were subcultured twice on nutrient agar before use in this study.

The original collection of these isolates was approved by the Ethics Committee of the Faculty of Pharmacy, Ain Shams University under approval numbers: (i) No. 212 for the studies by Elshamy et al. (2018) and Elshamy et al. (2020); and (ii) No. ENREC-ASU-2019–98 for the study by Elshamy et al. (2021). Written informed consent was obtained from all patients or their legal guardians during the original collection period, as detailed in the respective publications.

The present study involved only the *in vitro* analysis of previously collected bacterial isolates and did not involve any direct or indirect interaction with human subjects. Therefore, the Ethics Committee of the Faculty of Pharmacy, Ain Shams University, determined that no new ethical approval was required for this study.

### 2.2 Antibiogram of *K. pneumoniae*

A panel of 17 antibiotics representing diverse antimicrobial classes was employed in this study. Minimum inhibitory concentrations (MICs) were evaluated and interpreted according to CLSI guidelines (CLSI, 2025). Negative control wells containing sterile culture medium were included to ensure the absence of contamination, while positive control wells were inoculated with the reference quality control strain *Escherichia coli* ATCC 25922. After 24 hours of incubation at 37 °C, each well received 30 µL of 0.015% resazurin solution (Thermo Fisher Scientific, Waltham, MA, USA), and the plates were then incubated for an additional two hours. Resazurin’s color shift from blue to pink was thought to be a sign of bacterial growth (Costa et al., 2021).

### 2.3 Biofilm formation assay

Biofilm formation was assessed using the microtiter plate assay (O'Toole, 2011), with minor modifications. Briefly, bacterial isolates were grown overnight in trypticase soy broth (TSB; HiMedia, Mumbai, India). The cultures were adjusted to a turbidity equivalent to a 0.5 McFarland standard by spectrophotometric measurement at 600 nm and subsequently diluted 1:20 in fresh TSB. In duplicate wells of sterile, flat-bottom 96-well microtiter plates (Corning, NY, USA), 200 µL of the diluted suspension was added. The plates were then statically incubated at 37 °C for 24 hours. To get rid of non-adherent bacteria, the wells were gently rinsed with 200 µL of sterile phosphate-buffered saline (PBS) after the planktonic cells were carefully removed. After that, the plates were allowed to air dry for fifteen minutes at room temperature. 200 µL of 0.1% (v/v) crystal violet was used to fix and stain the adhering biofilms for 15 minutes. After using PBS to wash away any remaining discoloration, the plates were allowed to dry. After adding 200 µL of 33% (v/v) acetic acid and letting it sit at 37 °C for 15 minutes, the retained dye was dissolved. A microplate reader (ELx800, BioTek, Winooski, VT, USA) was used to measure the optical density (OD) at 630 nm to quantify biofilm biomass. *K. pneumoniae* ATCC 43816 was used as a positive control, while wells containing sterile media without inoculation were used as negative controls. The mean OD values were used to express the findings of each experiment, which was conducted in triplicate. The cutoff optical density (ODc) was calculated as the mean OD of the negative control plus three standard deviations (ODc = OD_nc + 3 × SD_nc). Based on OD values relative to ODc, isolates were classified as: non-biofilm producers (OD ≤ ODc), weak producers (ODc< OD ≤ 2×ODc), moderate producers (2×ODc< OD ≤ 4×ODc), or strong biofilm producers (OD > 4×ODc) (Majidi Fard et al., 2025).

### 2.4 Extraction of octadecyl 3-(3, 5-di-tert-butyl-4-hydroxyphenyl) propanoate

ODHP was produced, optimized, extracted, and purified from the culture broth of *Alcaligenes faecalis*, as previously described (El-Sayed et al., 2020). Briefly, the cell-free supernatant was extracted with ethyl acetate, and the crude extract was subjected to silica gel column chromatography using linear gradients of chloroform and ethyl acetate with increasing polarity. Fractions were collected, pooled based on thin-layer chromatography profiles, and tested for antifungal activity by bioautography against *Candida albicans* ATCC 10231. The purified compound was identified by UV absorption and NMR spectroscopy (¹H, ¹³C, COSY, HSQC, and HMBC) as octadecyl 3-(3,5-di-tert-butyl-4-hydroxyphenyl) propanoate (El-Sayed et al., 2020). The producing strain, *Alcaligenes faecalis* MT332429, was previously identified based on 16S rRNA gene sequencing and is available in the NCBI GenBank database under accession number MT332429 (accessed June 22, 2025). The isolate was also deposited in the Culture Collection Ain Shams University (CCASU) belonging to the World Data Centre for Microorganisms (WDCM) under the code, *Alcaligenes faecalis* CCASU-MT332429 (https://doi.org/10.12210/ccinfo.1186).

### 2.5 Antibacterial activities of ODHP

A stock solution of ODHP (1024 µg/mL) was initially prepared by dissolving the compound in dimethyl sulfoxide (DMSO) as previously described (El-Sayed et al., 2020). The final concentration of DMSO in the test system did not exceed 1% (v/v). The antibacterial activity of ODHP against MDR *K. pneumoniae* was evaluated as previously reported (Fanoro and Oluwafemi, 2025). Positive control wells contained bacterial suspension in CAMHB with the corresponding concentration of solvent but without ODHP, while negative control wells consisted of sterile medium without bacterial inoculation.

### 2.6 ODHP-loaded nanosponges preparation

ODHP-loaded nanosponges (NS) were prepared by the emulsion solvent diffusion method as previously described (El-Sayed et al., 2024). Briefly, different molar ratios of β-cyclodextrin and diphenyl carbonate (cross-linker) were dissolved in dichloromethane with 105 µg ODHP to form the dispersed phase. The continuous phase was prepared by dissolving 0.5% w/v polyvinyl alcohol (PVA) in distilled water at 60 °C. The dispersed phase was slowly injected into the continuous phase using a syringe while the mixture was emulsified by probe sonication at 35 °C for 10 min, then homogenized. The resulting nanosponges were filtered through 0.45-µm filter paper, washed with deionized water and ethanol/methanol (50:50), pre-frozen at -80 °C for 12 h, and lyophilized at -48 °C and 0.37 mbar for 2 days using mannitol as a cryoprotectant. The formulation was optimized using a Box-Behnken design with particle size, polydispersity index (PDI), and entrapment efficiency (EE%) as dependent variables (El-Sayed et al., 2024). The ODHP-loaded nanosponges were investigated for their antibacterial activity against MDR *K. pneumoniae* in comparison to ODHP.

### 2.7 Antibiofilm activity of ODHP and ODHP-NS concentrations

The antibiofilm activity of ODHP and its nanosponge formulation (ODHP-NS) was assessed at sub-inhibitory concentrations using the microtiter plate assay, as previously described (Abdullah and Younis, 2024), with minor modifications. The percentage of biofilm inhibition was calculated according to Shao et al. using the following equation (Shao et al., 2015):


$$\text{Biofilm inhibition }\left(\%\right)=\frac{\text{Ac}-\text{As}}{\text{Ac}}\times 100$$

where “Ac” represents the OD_630_ of untreated control wells, and “As” represents the OD_630_ of treated wells.

### 2.8 Growth rate analysis

The growth kinetics of selected isolates, including strong biofilm-forming strains, weak biofilm-forming strains, and *K. pneumoniae* ATCC 43816, were evaluated. For each strain, 20 µL of an overnight culture adjusted to 0.5 McFarland standard was inoculated into 180 µL of TSB in sterile 96-well microtiter plates. The plates were incubated at 37 °C, and bacterial growth was monitored by measuring the optical density at 600 nm (OD600) using a microplate reader (ELx800, BioTek, Winooski, VT, USA) at 4 h intervals over a period of 48 h (Chakraborty et al., 2025). All experiments were conducted in triplicate, and results were expressed as mean OD values. In parallel, the effect of sub-inhibitory concentrations (1/2 and 1/4 MICs) of ODHP and ODHP-NS on bacterial growth was assessed using the same experimental conditions. Growth inhibition was determined by comparing OD600 values of treated cultures with those of untreated controls. All experiments were performed in triplicate using untreated cultures as growth controls.

### 2.9 Synergistic activity of ODHP-NS in combination with ceftriaxone

The potential synergistic antibacterial activity between ODHP-NS, ODHP, and ceftriaxone (CRO) was evaluated using the standard broth microdilution checkerboard assay as previously described, with slight modifications (Bellio et al., 2021). Briefly, two-fold serial dilutions of ODHP, ODHP-NS, and ceftriaxone were prepared in CAMHB within sterile 96-well microtiter plates to generate a two-dimensional concentration matrix (checkerboard format). Each antimicrobial agent was tested individually and in combination across a range of concentrations surrounding their respective MICs. Bacterial inocula were prepared from overnight cultures of *K. pneumoniae* isolates and adjusted to a turbidity equivalent to 0.5 McFarland standard, followed by dilution to achieve a final concentration of approximately 5 × 10^5^ CFU/mL in each well. Plates were incubated at 37 °C for 18–24 h under aerobic conditions (Atta et al., 2023). Following incubation, bacterial growth was assessed visually or using a colorimetric indicator, and the MIC values of each agent alone and in combination were determined. The interaction between ODHP-NS and ceftriaxone was quantified by calculating the fractional inhibitory concentration index (FICI) using the following equation (Donkor et al., 2023):


$$\text{FICI}=\frac{\text{MIC}\_\text{ODHP}-\text{NS}\_\text{combination }}{\text{MIC}\_\text{ODHP}-\text{NS}\_\text{alone}}+\frac{\text{MIC}\_\text{CRO}\_\text{combination }}{\text{MIC}\_\text{CRO}\_\text{alone}}$$

FICI values were interpreted as synergistic (≤0.5), additive (>0.5–1), indifferent (>1–4), or antagonistic (>4) (Yang et al., 2021). All experiments were performed in triplicate on three separate occasions (biological replicates) for each combination tested to ensure reproducibility. The modal FICI value from the three independent experiments is reported.

### 2.10 Time-kill assay

To confirm the synergistic interactions observed in the checkerboard assay, selected combinations were further evaluated using time-kill kinetics, where a ≥2 log_10_ reduction in bacterial counts compared to the most active single agent was considered indicative of synergy (Zhou et al., 2020).

### 2.11 Statistical analysis

All statistical analyses were performed using the R statistical environment within RStudio (version 4.4.1). Data processing, analysis, and visualization were conducted using several R packages, including *readxl*, *ggplot2*, *ggpubr*, *tidyverse*, and *polycor*. The normality of quantitative variables was assessed before analysis. For normally distributed data, comparisons between two groups and among multiple groups were carried out using Student’s *t*-test and one-way analysis of variance (ANOVA), respectively. For non-normally distributed data, the Kruskal–Wallis test was employed to compare group medians. *Post hoc* analyses were performed using the Mann–Whitney *U* test following Kruskal–Wallis analysis and Tukey’s honestly significant difference (HSD) test following ANOVA. Bonferroni correction was applied to adjust for multiple comparisons where appropriate. Correlations were assessed using Spearman’s rank correlation coefficient (*r*_s_), with values ranging from −1 to +1 to indicate the strength and direction of associations.

## 3 Results

### 3.1 Antibiogram of *K. pneumoniae* isolates and resistance analysis

**Figure 1:**
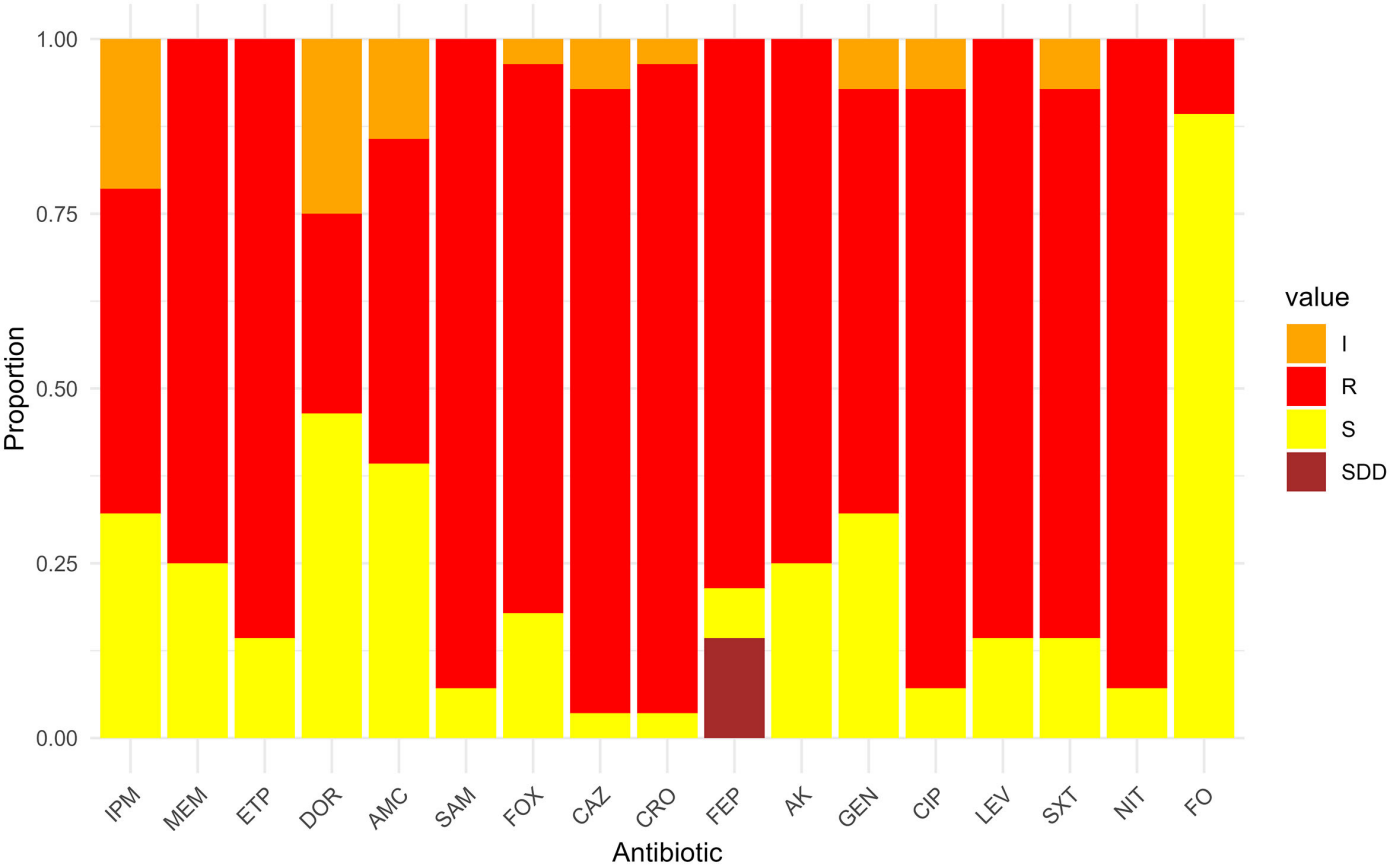
Distribution of antimicrobial susceptibility patterns among *Klebsiella pneumoniae* isolates. The stacked bar chart represents the proportion of isolates categorized as susceptible (S), intermediate (I), resistant (R), and susceptible dose-dependent (SDD) for each tested antibiotic.

The antimicrobial susceptibility patterns of the tested *K. pneumoniae* isolates are presented in **Figure 1** as proportional stacked bar plots. Overall, a high prevalence of resistance was observed across most antibiotic classes, indicating a multidrug-resistant phenotype among the isolates. Carbapenems demonstrated variable activity, with imipenem (IPM) and meropenem (MEM) showing partial susceptibility; however, resistance remained predominant. Notably, ertapenem (ETP) and doripenem (DOR) exhibited higher resistance rates compared to other carbapenems. Among β-lactam/β-lactamase inhibitor combinations, ampicillin/sulbactam (SAM) showed extensive resistance, whereas amoxicillin/clavulanate (AMC) displayed comparatively improved susceptibility, although resistance still dominated. Cephalosporins, including ceftazidime (CAZ), ceftriaxone (CRO), and cefepime (FEP), were largely ineffective, with resistance observed in the majority of isolates. Aminoglycosides demonstrated moderate activity, with gentamicin (GEN) showing relatively higher susceptibility compared to amikacin (AK), although resistance was still substantial. Similarly, fluoroquinolones (ciprofloxacin (CIP) and levofloxacin (LEV)) exhibited high resistance rates, indicating limited therapeutic effectiveness.

Among the remaining agents, trimethoprim/sulfamethoxazole (SXT) and nitrofurantoin (NIT) showed predominantly resistant profiles. In contrast, fosfomycin (FO) exhibited the highest susceptibility among all tested antibiotics, with the majority of isolates classified as sensitive and minimal resistance observed. Collectively, these findings highlight the extensive antimicrobial resistance among the tested isolates, with fosfomycin emerging as the most effective agent, while most β-lactams and fluoroquinolones demonstrated limited activity. The resistance profiles of *K. pneumoniae* isolates against the tested antibiotics are illustrated in a hierarchical clustered heatmap (**Supplementary Figure 1**). A high prevalence of resistance was observed across the majority of antibiotics, as indicated by the dominance of red coloration. Hierarchical clustering revealed distinct groups of isolates sharing similar resistance patterns, suggesting the presence of common resistance determinants. Several isolates exhibited resistance to nearly all tested antibiotics, confirming a multidrug-resistant phenotype. In contrast, limited susceptibility was observed for certain antibiotics, reflected by the presence of sensitive (yellow) and intermediate (orange) responses in a subset of isolates. These findings highlight the heterogeneity of resistance patterns among the studied isolates while emphasizing the widespread occurrence of multidrug resistance.

### 3.2 Correlation analysis of antibiotic resistance

**Figure 2:**
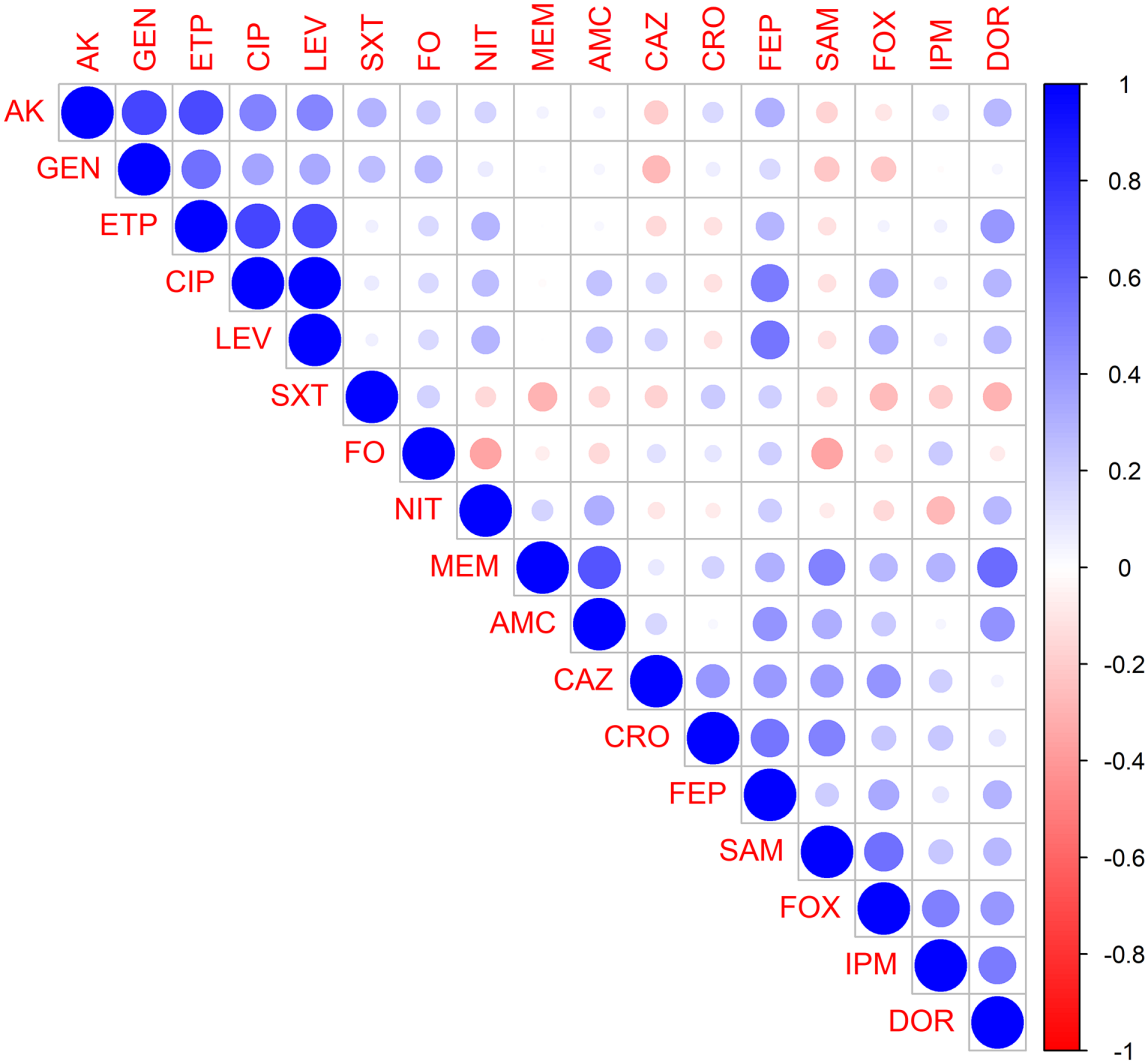
Correlation matrix of antimicrobial resistance profiles among tested antibiotics in *K. pneumoniae* isolates. Spearman’s rank correlation coefficients (r_s_) are represented by circle size and color intensity, where blue indicates positive correlations and red indicates negative correlations. Strong positive correlations were observed among antibiotics within the same class, indicating co-resistance patterns.

The correlation matrix of antimicrobial resistance patterns among the tested antibiotics is presented in **Figure 2**. Spearman’s rank correlation analysis revealed distinct clustering patterns, indicating shared resistance mechanisms among several antibiotic classes. Strong positive correlations (r_s_ close to +1) were observed among β-lactam antibiotics, particularly between cephalosporins (CAZ, CRO, FEP) and β-lactam/β-lactamase inhibitor combinations (AMC, SAM, FOX), suggesting co-resistance likely mediated by common mechanisms such as extended-spectrum β-lactamase (ESBL) production. Additionally, carbapenems (IPM, MEM, DOR, and ETP) exhibited moderate to strong positive correlations with each other, indicating possible cross-resistance within this class. Aminoglycosides (AK and GEN) also demonstrated strong positive correlations, reflecting similar resistance determinants. Likewise, fluoroquinolones (CIP and LEV) showed a strong positive association, consistent with shared mechanisms such as target site mutations or efflux pump overexpression. In contrast, several weak or negative correlations (r_s_ close to 0 or negative values) were observed between unrelated antibiotic classes, such as between fosfomycin (FO), nitrofurantoin (NIT), and trimethoprim/sulfamethoxazole (SXT) versus β-lactams and carbapenems. These findings suggest that resistance to these agents may arise from independent mechanisms and may not be co-selected with β-lactam resistance. Overall, the correlogram highlights the presence of multidrug resistance clusters among *K. pneumoniae* isolates, with strong intra-class correlations and selective inter-class associations, providing insights into potential co-resistance and cross-resistance patterns.

### 3.3 Antibacterial activity of ODHP and ODHP-NS (agar diffusion assay)

**Figure 3:**
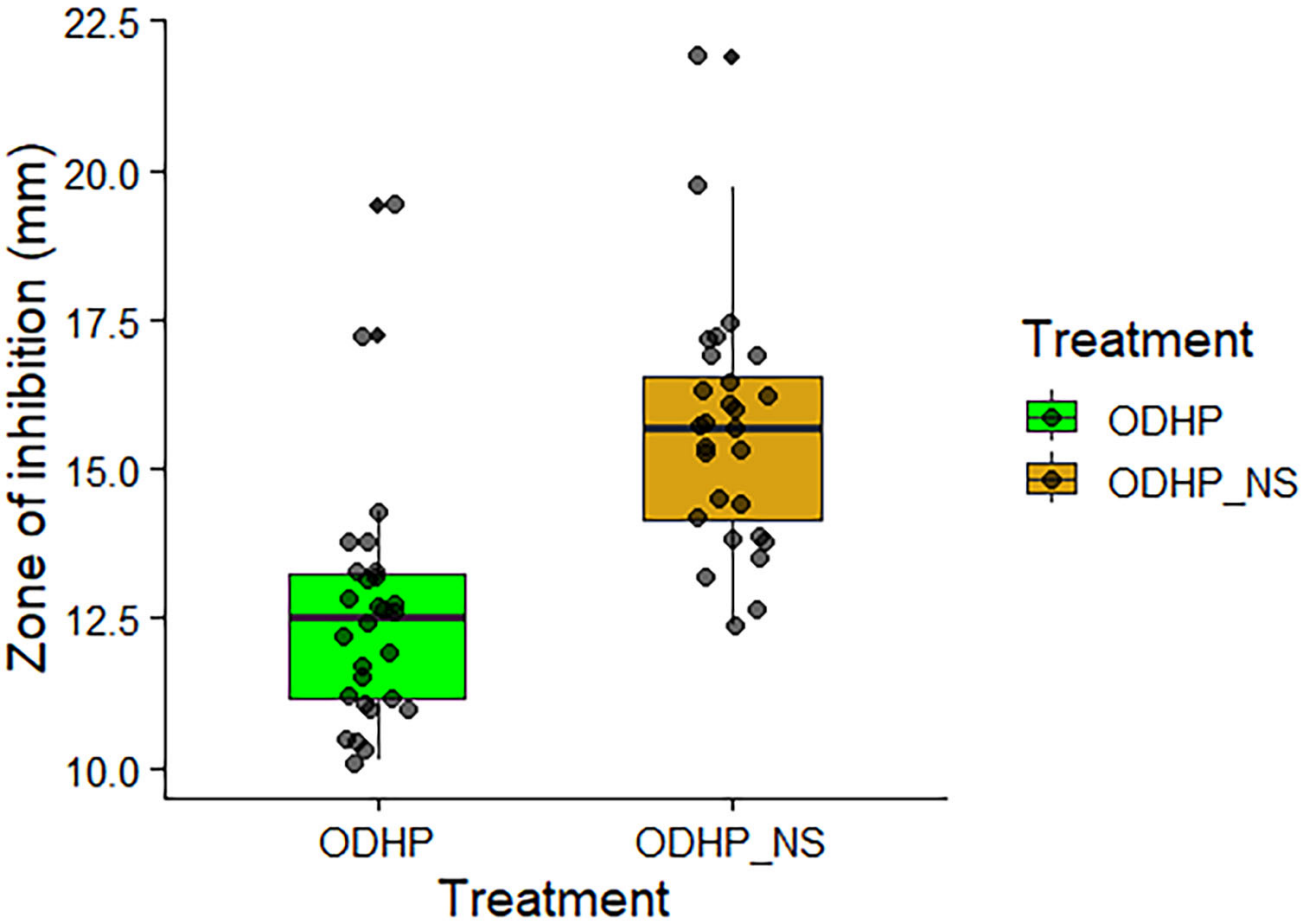
Comparative analysis of inhibition zones produced by ODHP and ODHP-NS against *K. pneumoniae* isolates. Boxplots represent the distribution of inhibition zone diameters (mm), with individual data points overlaid. ODHP-NS exhibited significantly larger inhibition zones compared to ODHP (p< 0.05, Wilcoxon signed-rank test), indicating enhanced antibacterial activity following nanosponge formulation.

The antibacterial activity of ODHP and ODHP-NS against *K. pneumoniae* isolates was evaluated using the agar diffusion method, and the results are presented in **Figure 3**. As ODHP is an investigational compound without established CLSI breakpoints, inhibition zone diameters were compared directly between ODHP and ODHP-NS using the same assay conditions. The statistical significance of the difference between the two treatments was assessed using the Wilcoxon signed-rank test.

ODHP-NS exhibited larger zones of inhibition compared to ODHP across the tested isolates. The median inhibition zone for ODHP-NS was higher than that of ODHP, with most ODHP-NS values ranging between approximately 14 and 17 mm. In contrast, ODHP inhibition zones were generally lower, predominantly distributed between approximately 11 and 13 mm. The distribution of inhibition zones for ODHP-NS was shifted toward higher values, with several isolates showing zones exceeding 18 mm. ODHP displayed a narrower distribution with lower overall values. Individual data points further indicate that, for the majority of isolates, ODHP-NS produced greater inhibition zones than ODHP. A statistically significant difference was observed between ODHP and ODHP-NS treatments (*p* < 0.05).

### 3.4 MIC distribution and comparative antimicrobial activity

The MIC distributions of the tested antibiotics, ODHP, and ODHP-NS against *K. pneumoniae* isolates (n = 28) are summarized in **Table 1**. Ceftriaxone (CRO) exhibited the highest MIC values among the tested antibiotics, with both MIC_50_ and MIC_90_ recorded at 512 µg/mL. Cefepime (FEP) also demonstrated elevated MIC values (MIC_50_ = 96 µg/mL; MIC_90_ = 256 µg/mL), with a wide distribution across tested concentrations. In contrast, meropenem (MEM) showed the lowest MIC values, with a median MIC (MIC_50_) of 2.5 µg/mL and MIC_90_ of 64 µg/mL. Gentamicin (GEN) and ciprofloxacin (CIP) exhibited intermediate MIC distributions, with MIC_50_ values of 32 µg/mL and MIC_90_ values of 512 µg/mL and 256 µg/mL, respectively. ODHP demonstrated MIC_50_ and MIC_90_ values of 96 µg/mL and 256 µg/mL, respectively. In comparison, ODHP-NS exhibited lower MIC values, with MIC_50_ = 32 µg/mL and MIC_90_ = 128 µg/mL. The MIC distribution of ODHP-NS was shifted toward lower concentrations, with no isolates exhibiting MIC values ≥256 µg/mL. Statistical analysis indicated that ODHP-NS exhibited significantly lower MIC values compared to CRO (*p* < 0.001), FEP (*p* < 0.01), GEN (*p* < 0.01), and CIP (*p* < 0.01), as well as ODHP (*p* < 0.05).

**Table 1:** Minimum inhibitory concentration (MIC) distribution (µg/mL) of selected antibiotics, ODHP, and ODHP-NS against *K. pneumoniae* isolates.

Antibiotic	≤1	2	4	8	16	32	64	128	256	512	MIC_50_ (µg/mL)	MIC_90_ (µg/mL)	*p*-value vs ODHP-NS
CRO	0	2	0	0	0	0	2	0	6	18	512	512	<0.001
FEP	0	2	0	0	2	4	6	6	6	2	96	256	<0.01
MEM	9	0	1	4	1	1	3	2	2	0	2.5	64	ns
GEN	4	0	4	0	2	6	4	0	0	8	32	512	<0.01
CIP	4	2	0	2	0	12	2	2	2	2	32	256	<0.01
ODHP	0	0	1	1	2	3	2	7	6	1	96	256	<0.05
ODHP-NS	0	2	0	3	2	4	5	7	0	0	32	128	—

P-values were calculated using appropriate statistical tests, with *p* < 0.05 considered significant.

CRO, ceftriaxone; FEP, cefepime; MEM, meropenem; GEN, gentamicin; CIP, ciprofloxacin; ODHP, octadecyl 3-(3,5-di-tert-butyl-4-hydroxyphenyl) propanoate; ODHP-NS, octadecyl 3-(3,5-di-tert-butyl-4-hydroxyphenyl) propanoate nanosponge; ns, not significant.

The *p*-value for ODHP represents the statistical comparison between ODHP and ODHP-NS.

MIC_50_ and MIC_90_ values are presented, along with statistical comparisons between ODHP and ODHP-NS.

### 3.5 Heatmap analysis of MIC profiles

**Figure 4:**
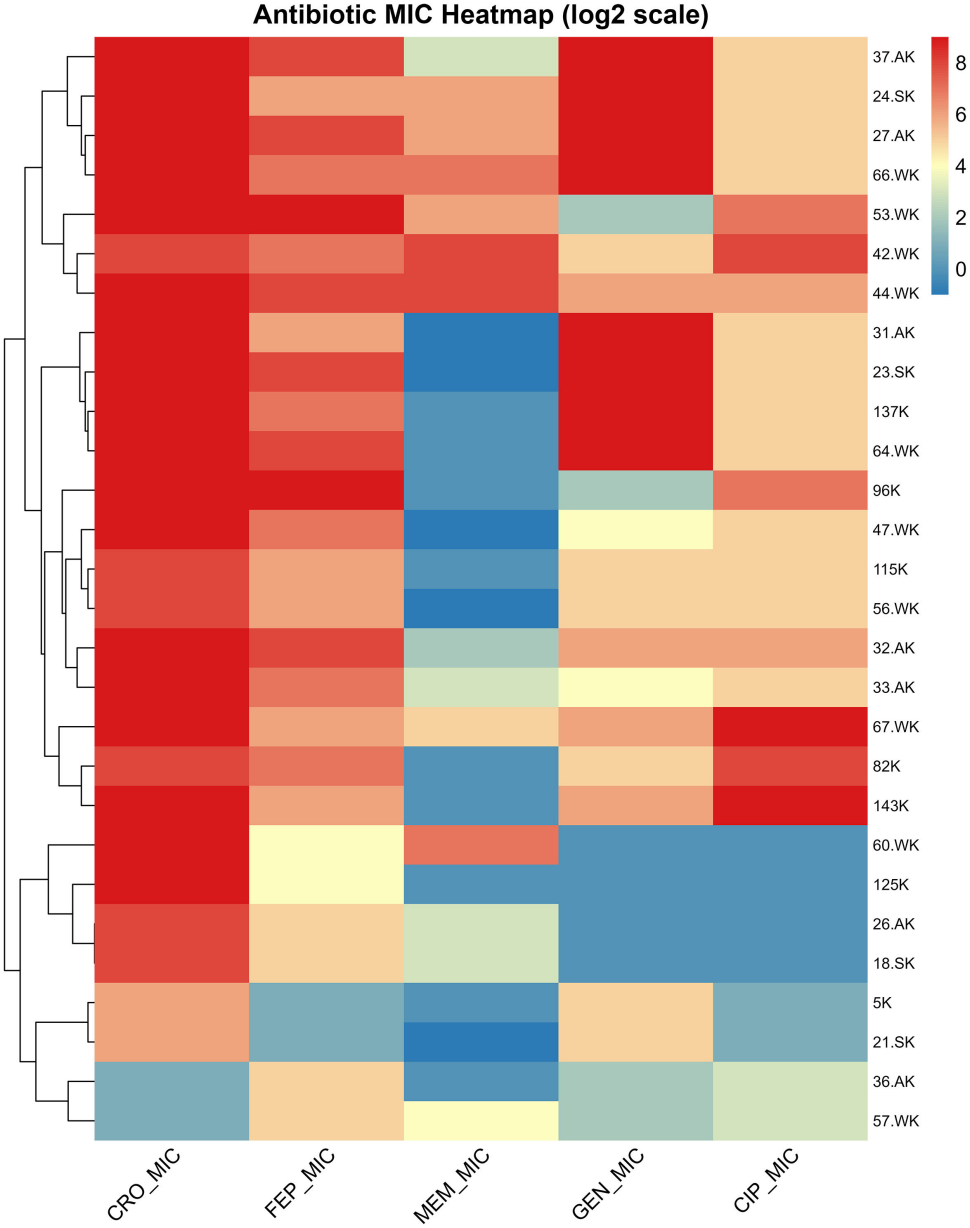
Heatmap of minimum inhibitory concentrations (MICs) for selected antibiotics against *K. pneumoniae* isolates. MIC values were log_2_-transformed and visualized using a color gradient (blue = low MIC, red = high MIC). Hierarchical clustering (Euclidean distance, complete linkage) reveals distinct patterns among isolates and clustering of antibiotics with similar activity profiles.

The overall distribution of MIC values across isolates is presented as a hierarchical clustered heatmap (**Figure 4**). MIC values were log_2_-transformed prior to visualization to facilitate comparison across a wide concentration range. The heatmap demonstrates substantial variability in MIC values among isolates. CRO and FEP exhibited consistently high MIC values across most isolates, as indicated by dominant red coloration. In contrast, MEM displayed lower MIC values, reflected by blue to light-colored regions. GEN and CIP showed heterogeneous MIC distributions, with both low and high values observed across isolates. Hierarchical clustering revealed distinct isolate groups based on MIC profiles. One cluster was characterized by consistently elevated MIC values across multiple antibiotics, while another cluster exhibited comparatively lower MIC values, particularly for MEM and GEN. Clustering of antibiotics demonstrated close grouping between CRO and FEP, whereas MEM formed a separate cluster corresponding to lower MIC values.

### 3.6 Comparative MIC distribution of ODHP and ODHP-NS

**Figure 5:**
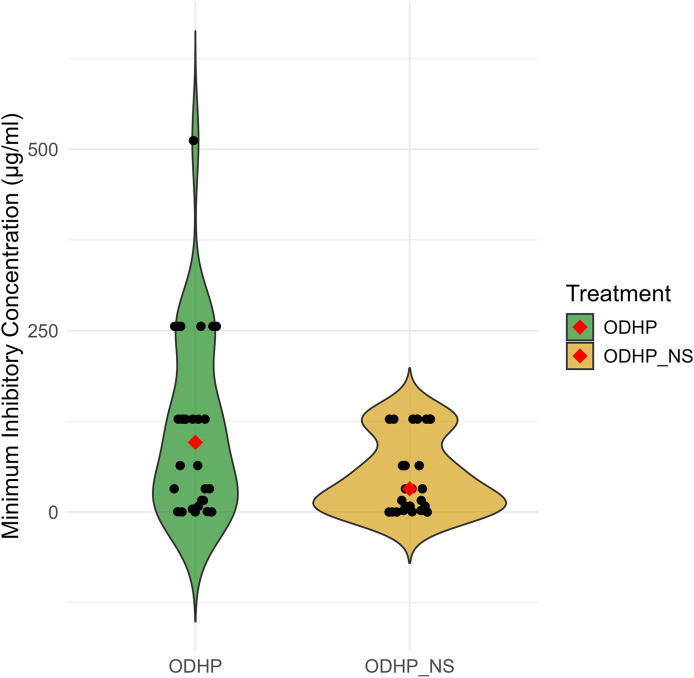
Distribution of minimum inhibitory concentrations (MICs) of ODHP and ODHP-NS against *K. pneumoniae* isolates. Violin plots illustrate the distribution and density of MIC values, with overlaid points representing individual isolates. ODHP-NS demonstrated lower MIC values compared to ODHP, indicating improved antimicrobial potency.

The distribution of MIC values for ODHP and ODHP-NS is shown in **Figure 5**. Raw MIC values were used for this analysis. ODHP-NS exhibited a distribution shifted toward lower MIC values compared to ODHP. The majority of ODHP-NS MIC values were concentrated between approximately 16 and 128 µg/mL, whereas ODHP displayed a broader distribution extending to higher concentrations, including values of 256 µg/mL and above. The median MIC value for ODHP-NS was lower than that of ODHP. ODHP also showed greater variability, with a wider spread of MIC values. Individual data points indicate that most isolates exhibited lower MIC values when treated with ODHP-NS compared to ODHP. Statistical analysis using the Wilcoxon signed-rank test demonstrated a significant difference between ODHP and ODHP-NS MIC values (*p* < 0.05).

### 3.7 Association between resistance burden and biofilm formation

**Figure 6:**
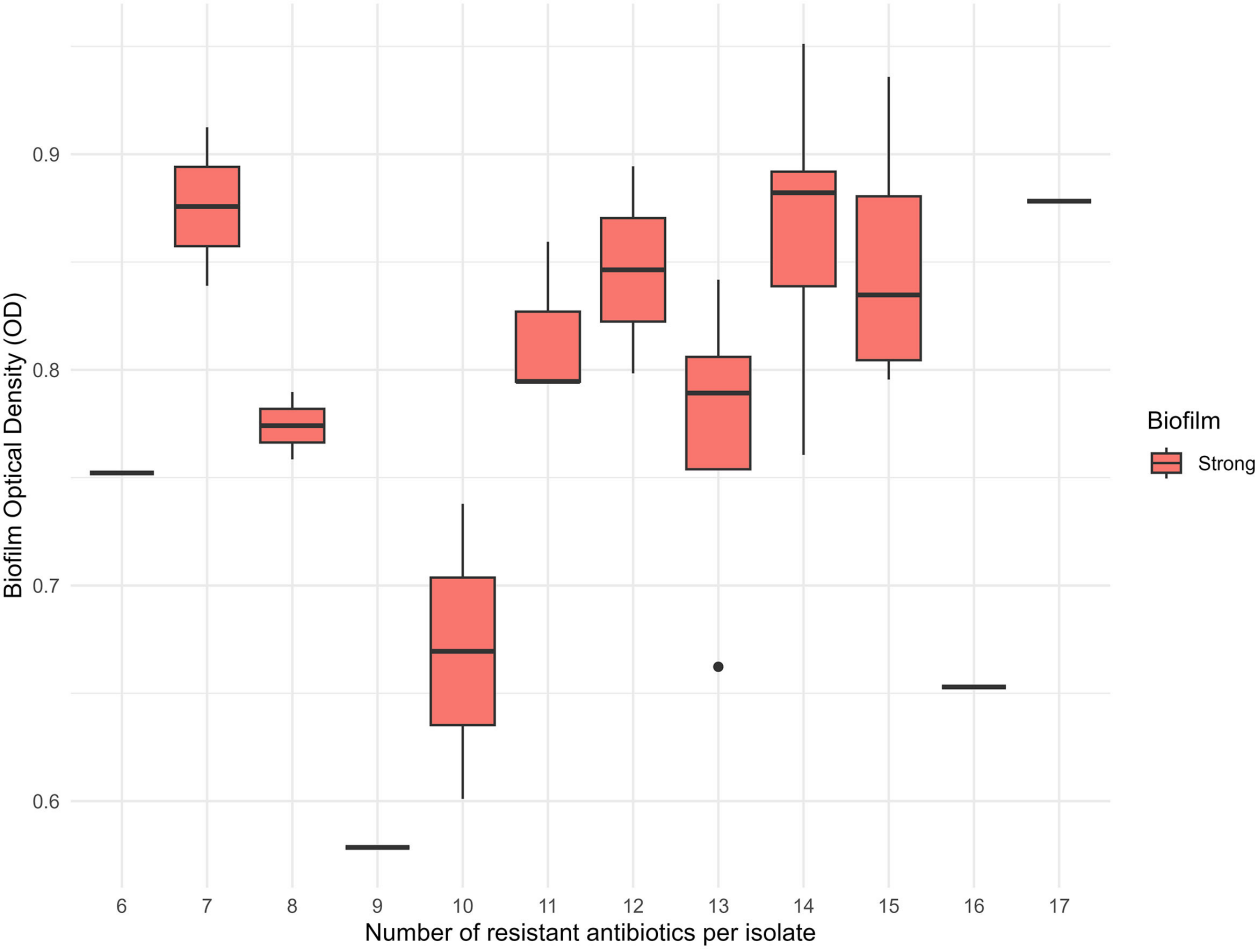
Relationship between biofilm formation and antimicrobial resistance burden among *K. pneumoniae* isolates. Boxplots represent biofilm optical density (OD) values across isolates grouped by the number of antibiotics they were resistant to. A significant trend toward higher biofilm formation with increased resistance burden was observed (Fisher’s exact test, *p* < 0.05).

The distribution of biofilm-forming capacity across resistance clusters of *K. pneumoniae* isolates is summarized in **Table 2** and illustrated in **Figure 6**. Isolates were grouped into six clusters according to the number of antibiotics to which they were resistant. As shown in **Table 2**, isolates with higher resistance profiles demonstrated a greater proportion of strong biofilm formation. In cluster I (≥5 resistant antibiotics), 66.7% of isolates were classified as strong biofilm producers, compared to 25.0% and 8.3% categorized as moderate and weak, respectively. Similarly, cluster II (4 resistant antibiotics) showed 57.1% strong, 28.6% moderate, and 14.3% weak biofilm producers. In cluster III (3 resistant antibiotics), strong and moderate biofilm formation were equally represented (42.9% each), with 14.3% weak producers. In cluster IV (2 resistant antibiotics), moderate biofilm formation predominated (42.9%), followed by equal proportions of strong and weak producers (28.6% each). A shift toward weaker biofilm formation was observed in clusters with lower resistance. In cluster V (1 resistant antibiotic), weak biofilm producers accounted for 50.0% of isolates, followed by moderate (33.3%) and strong (16.7%) categories. In cluster VI (no resistance), no isolates were classified as strong biofilm producers, while 25.0% and 75.0% were moderate and weak producers, respectively. These findings are consistent with the distribution of optical density (OD) values presented in **Figure 6**. The boxplot demonstrates variability in biofilm biomass across isolates with different resistance burdens, with OD values generally ranging between approximately 0.60 and 0.95. Higher OD values were observed among isolates resistant to a greater number of antibiotics, while lower OD values were observed among isolates resistant to fewer antibiotics. Overall, the combined table and figure demonstrate variation in biofilm formation across resistance clusters, with differences in both categorical distribution and quantitative OD measurements. Statistical analysis (Fisher’s exact test) demonstrated a significant association between resistance clusters and biofilm formation categories (*p* < 0.05).

**Table 2:** Association between antimicrobial resistance burden and biofilm formation among *K. pneumoniae* isolates.

Cluster (No. of antibiotics to which isolates were resistant)	Strong (n, %)	Moderate (n, %)	Weak (n, %)
I (≥5)	8 (66.7%)	3 (25.0%)	1 (8.3%)
II (4)	4 (57.1%)	2 (28.6%)	1 (14.3%)
III (3)	3 (42.9%)	3 (42.9%)	1 (14.3%)
IV (2)	2 (28.6%)	3 (42.9%)	2 (28.6%)
V (1)	1 (16.7%)	2 (33.3%)	3 (50.0%)
VI (0)	0 (0%)	1 (25.0%)	3 (75.0%)

Isolates were grouped based on the number of antibiotics to which they were resistant, and biofilm formation was categorized as strong, moderate, or weak. Data are presented as numbers and percentages.

### 3.8 Antibiofilm activity of ODHP and ODHP-NS

**Figure 7:**
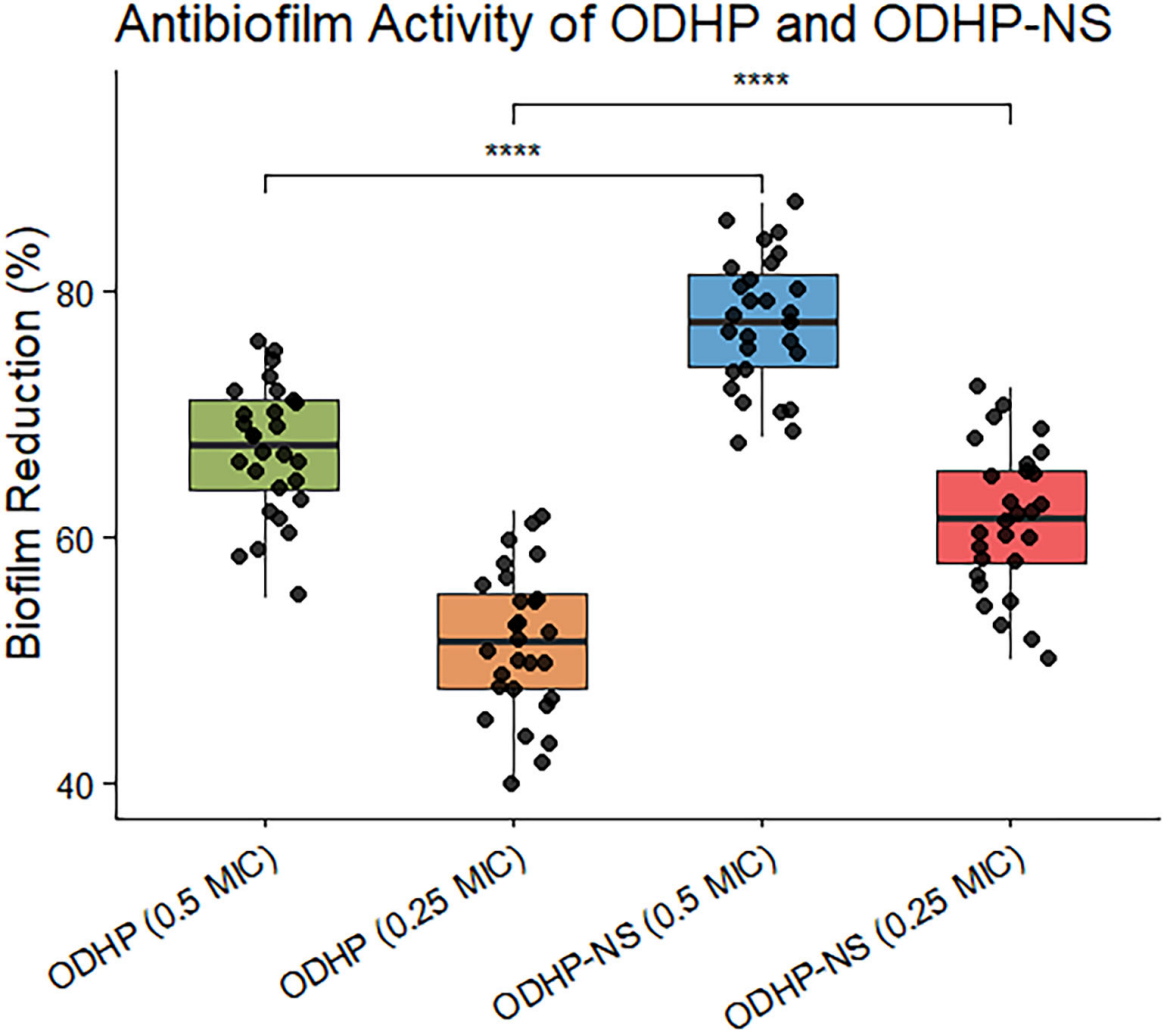
Antibiofilm activity of ODHP and ODHP-NS at sub-inhibitory concentrations (1/2 MIC and 1/4 MIC). Boxplots represent the percentage reduction in biofilm formation, with individual data points shown. ODHP-NS exhibited significantly higher biofilm inhibition compared to ODHP across both concentrations.

The antibiofilm activity of ODHP and ODHP-NS was evaluated at sub-inhibitory concentrations (1/2 MIC and 1/4 MIC) against *K. pneumoniae* isolates, and the results are presented in **Figure 7**. Both ODHP and ODHP-NS demonstrated concentration-dependent inhibition of biofilm formation. At 1/2 MIC, ODHP exhibited higher biofilm reduction compared to its 1/4 MIC concentration, with median inhibition values centered around approximately 65–70%. Similarly, ODHP-NS at 1/2 MIC showed the highest antibiofilm activity among all tested conditions, with inhibition values predominantly ranging between approximately 75% and 85%. At 1/4 MIC, both treatments showed reduced antibiofilm activity compared to their respective 1/2 MIC concentrations. ODHP at 1/4 MIC exhibited lower inhibition values, primarily between approximately 45% and 55%, whereas ODHP-NS at 1/4 MIC showed moderate activity, with inhibition values generally between approximately 55% and 70%. Comparative analysis revealed that ODHP-NS exhibited significantly higher antibiofilm activity than ODHP at both tested concentrations. Statistical analysis using the Wilcoxon test confirmed that the differences between ODHP and ODHP-NS at 1/2 MIC and 1/4 MIC were highly significant (*p* < 0.0001). Overall, ODHP-NS demonstrated superior antibiofilm efficacy compared to ODHP across all tested conditions.

### 3.9 Synergistic activity of ODHP-NS in combination with ceftriaxone

The interaction between ODHP-NS and ceftriaxone (CRO) was evaluated using the checkerboard microdilution assay, and the results are summarized in **Table 3**.

**Table 3:** Checkerboard assay results showing the interaction between ODHP-NS and ceftriaxone.

ODHP-NS (µg/mL)	CRO (µg/mL)	FICI	Interpretation
16	256	0.75	Additive
32	128	0.5	Synergy
64	64	0.5	Synergy
32	256	0.75	Additive
64	128	0.5	Synergy

Fractional inhibitory concentration index values were calculated and interpreted as synergistic (≤0.5), additive (>0.5–1), indifferent (>1–4), or antagonistic (>4).

ODHP-NS, octadecyl 3-(3,5-di-tert-butyl-4-hydroxyphenyl) propanoate nanosponge; CRO, ceftriaxone; FICI, Fractional inhibitory concentration index.

The full checkerboard matrix was tested; the combinations shown represent those where FICI values were at the synergy or additivity threshold (FICI ≤ 0.5 or FICI >0.5–1). The remaining combinations showed indifference (FICI > 1–4). No antagonism (FICI > 4) was observed.

The combination of ODHP-NS and CRO demonstrated predominantly synergistic to additive interactions against the tested *K. pneumoniae* isolates. Synergistic effects (FICI ≤ 0.5) were observed in the majority of tested combinations, particularly at concentrations of 32/128 µg/mL, 64/64 µg/mL, and 64/128 µg/mL for ODHP-NS/CRO, respectively. Additive interactions (FICI = 0.75) were observed at ODHP-NS/CRO combinations of 16/256 µg/mL and 32/256 µg/mL. No antagonistic interactions were detected in any of the tested combinations. Overall, the combination of ODHP-NS with ceftriaxone resulted in a consistent reduction in MIC values compared to each agent alone, indicating enhanced antibacterial activity when used in combination. These findings indicate that ODHP-NS potentiates the antibacterial activity of ceftriaxone against resistant *K. pneumoniae* isolates.

### 3.10 Time–kill kinetics of ODHP-NS in combination with ceftriaxone

**Figure 8:**
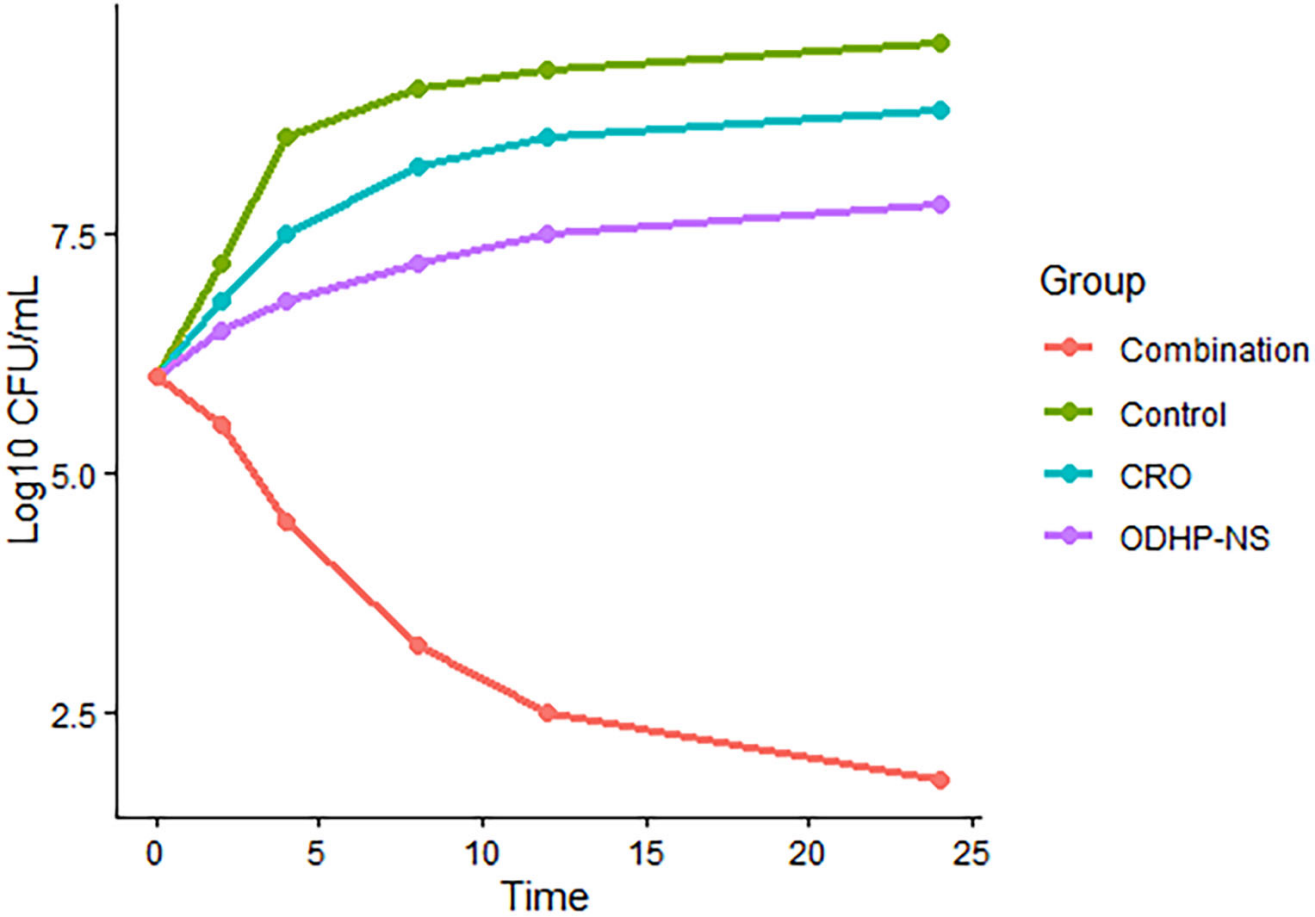
Time–kill kinetics of ODHP-NS, ceftriaxone (CRO), and their combination against *K. pneumoniae*. Bacterial counts (log_10_ CFU/mL) were measured over 24 h. The combination therapy demonstrated a marked reduction in bacterial counts compared to monotherapy and control, indicating synergistic bactericidal activity.

The bactericidal activity of ODHP-NS, ceftriaxone (CRO), and their combination against *K. pneumoniae* was evaluated using a time–kill assay over 24 h (**Figure 8**). The untreated control exhibited a continuous increase in bacterial growth, reaching approximately 9 log_10_ CFU/mL at 24 h. Similarly, treatment with ceftriaxone alone showed limited antibacterial activity, with bacterial counts increasing over time, although at a slightly reduced rate compared to the control. ODHP-NS alone demonstrated moderate antibacterial activity, resulting in partial suppression of bacterial growth; however, bacterial counts still increased over the incubation period, reaching approximately 7.5–8 log_10_ CFU/mL at 24 h. In contrast, the combination of ODHP-NS and ceftriaxone exhibited a marked bactericidal effect, with a rapid decline in bacterial counts observed as early as 4 h. The bacterial load decreased progressively over time, reaching approximately 2 log_10_ CFU/mL at 24 h. Notably, the combination treatment resulted in a reduction exceeding 3 log_10_ CFU/mL compared to the most active single agent, indicating a synergistic bactericidal interaction. No regrowth was observed throughout the incubation period.

## 4 Discussion

The present study investigated the antibacterial and antibiofilm activities of octadecyl 3-(3,5-di-tert-butyl-4-hydroxyphenyl) propanoate (ODHP) and its nanosponge formulation (ODHP-NS) against clinical MDR *K. pneumoniae* isolates, and explored the synergistic potential of ODHP-NS in combination with ceftriaxone. The results collectively demonstrate that nanosponge encapsulation markedly enhances the intrinsic antibacterial and antibiofilm properties of ODHP against MDR *K. pneumoniae*, and that ODHP-NS potentiates the activity of a clinically challenged β-lactam antibiotic (ceftriaxone) against resistant strains. The extensive antimicrobial resistance observed among our clinical *K. pneumoniae* isolates is consistent with the globally reported escalation of MDR in this pathogen. In the present study, high resistance rates were documented across most antibiotic classes, including cephalosporins, fluoroquinolones, and carbapenems, while fosfomycin retained the highest level of susceptibility. These findings align with data from systematic surveillance studies, which report pooled resistance prevalence exceeding 50% to first-line antibiotics and rising carbapenem resistance, particularly in resource-limited healthcare settings (Yakobi and Nwodo, 2025). The clinical challenge posed by MDR and carbapenem-resistant *K. pneumoniae* (CRKP) is well documented; mortality associated with CRKP infections remains alarmingly high, with carbapenem resistance ranging from 3–7% in Europe and the United States to over 20% in Asia and Latin America (Shah et al., 2025). The dominance of resistance to β-lactams, fluoroquinolones, and aminoglycosides in our isolates underscores the urgent need for novel antimicrobial strategies beyond conventional antibiotic classes.

Correlation analysis of resistance patterns revealed strong intra-class associations, particularly among cephalosporins, carbapenems, and aminoglycosides, suggesting shared resistance determinants such as ESBL production and efflux pump overexpression. This co-resistance pattern is consistent with the molecular epidemiology of MDR *K. pneumoniae*, where mobile genetic elements, including plasmids and integrons, facilitate horizontal gene transfer of resistance determinants (Huy, 2024). ESBL-producing strains demonstrate the ability to hydrolyze most β-lactams, which likely accounts for the near-universal cephalosporin resistance observed. The notable activity of fosfomycin in our isolates is clinically significant, as this agent acts through a distinct mechanism – inhibition of UDP-N-acetylglucosamine enolpyruvyl transferase (MurA) – that is not co-selected with β-lactam or fluoroquinolone resistance determinants (Javan et al., 2025).

A significant positive association between the number of resistant antibiotics and biofilm-forming capacity was observed in the present study, with isolates belonging to higher resistance clusters demonstrating greater proportions of strong biofilm producers. This finding is in agreement with an extensive body of literature linking antibiotic resistance and biofilm formation in *K. pneumoniae*. A 2024 review of the molecular mechanisms underlying biofilm formation and antibiotic resistance in *K. pneumoniae* reported that biofilm-producing strains accounted for 75% of clinical isolates and that MDR isolates exhibited significantly higher biofilm-forming capacity compared to non-MDR isolates (*p* < 0.05) (Li et al., 2024). Similarly, studies on wound isolates have demonstrated that virtually all ESBL-positive *K. pneumoniae* strains were capable of biofilm production, confirming a strong link between ESBL carriage and biofilm phenotype (Allami et al., 2024).

The biological basis for this correlation is multifactorial. Biofilm formation confers an additional layer of antibiotic tolerance by limiting drug penetration, creating metabolic gradients that reduce antibiotic efficacy, and inducing phenotypic adaptations in slow-growing subpopulations. In MDR *K. pneumoniae*, biofilm-associated resistance can increase antibiotic tolerance by up to 1,000-fold compared to planktonic cells (Folliero et al., 2021; Li et al., 2024). Conversely, efflux pumps overexpressed as part of resistance mechanisms also contribute to biofilm regulation, creating a reinforcing cycle between resistance and biofilm phenotypes. These observations highlight the dual therapeutic challenge presented by MDR biofilm-forming *K. pneumoniae* and support the need for agents capable of targeting both phenotypic states simultaneously, as demonstrated in the present study with ODHP-NS.

ODHP-NS exhibited significantly enhanced antibacterial activity against MDR *K. pneumoniae* isolates compared to the free compound ODHP, as evidenced by lower MIC_50_ (32 vs. 96 µg/mL) and MIC_90_ (128 vs. 256 µg/mL) values, as well as larger inhibition zones in the agar diffusion assay. The statistically significant improvement in antibacterial potency following nanosponge formulation is consistent with the well-established pharmacological advantages of nanosponge-based drug delivery systems, including enhanced aqueous solubility, increased bioavailability, improved stability, and sustained controlled release of encapsulated compounds (Gandhi et al., 2025; Koppula and Maddi, 2024). Nanosponges, particularly those based on cyclodextrin crosslinked networks, form inclusion and non-inclusion complexes with hydrophobic drug molecules, increasing their effective aqueous concentration and protecting them from degradation (Tiwari and Bhattacharya, 2022). The enhanced antibacterial activity of ODHP-NS is likely attributed to the increased solubilization of the hydrophobic ODHP molecule within the nanosponge matrix, facilitating improved interaction with the bacterial cell envelope. This mechanism has been previously demonstrated with other hydrophobic antimicrobial compounds, where encapsulation of thyme essential oil in β-cyclodextrin nanosponges improved aqueous solubility 15-fold and reduced the MIC by up to 29.4-fold (Rezaei et al., 2021). Similar improvements in antibacterial efficacy following nanoformulation have been reported for imipenem-loaded poly(ϵ-caprolactone) nanospheres, where the MIC against MDR *K. pneumoniae* decreased 8-fold, attributed to improved drug stability against β-lactamase-mediated degradation and enhanced bacterial membrane interaction. ODHP itself was previously identified as an antifungal metabolite of *Alcaligenes faecalis* MT332429, with its production and purification optimized through response surface methodology (El-Sayed et al., 2020). The present study extends its biological profile to include clinically relevant antibacterial activity against MDR *K. pneumoniae*, and demonstrates that nanosponge formulation, previously reported by El-Sayed et al. (2024), improves MIC values under clinical conditions. The shift in MIC distribution of ODHP-NS toward lower concentrations, with no isolates exhibiting MICs ≥256 µg/mL, compared to ODHP, where several isolates reached such levels, represents a clinical improvement in the therapeutic window of this compound. Both ODHP and ODHP-NS demonstrated concentration-dependent inhibition of biofilm formation at sub-inhibitory concentrations (1/2 and 1/4 MIC), with ODHP-NS consistently exhibiting significantly higher antibiofilm activity (*p* < 0.0001). At 1/2 MIC, ODHP-NS achieved a median biofilm inhibition of approximately 75–85%, compared to 65–70% for ODHP, while at 1/4 MIC, ODHP-NS retained notable efficacy (55–70%) compared to ODHP (45–55%). The higher antibiofilm activity of ODHP-NS at sub-inhibitory concentrations is particularly clinically relevant because sub-MIC concentrations of antimicrobials are commonly encountered at infection sites due to pharmacokinetic limitations and tissue penetration barriers (Muller et al., 2004).

The enhanced antibiofilm activity of ODHP-NS may be mechanistically linked to the improved aqueous dispersibility and sustained-release properties conferred by the nanosponge matrix. Controlled release of ODHP from the nanosponge scaffold can maintain drug concentrations at biofilm interfaces over extended periods, providing a more sustained suppression of biofilm initiation compared to the free compound. Furthermore, the nanoscale dimensions of the nanosponge particles may facilitate deeper penetration into the extracellular polymeric substance (EPS) matrix of developing biofilms, increasing the effective drug concentration at the biofilm surface and within its interior (Saha and Rafe, 2023). This is analogous to observations with polymeric nanoparticles and nanosponges loaded with antimicrobial agents, which have been shown to demonstrate enhanced biofilm penetration and inhibition compared to free drugs (Li et al., 2024). The ability to inhibit biofilm at sub-inhibitory concentrations is of particular importance given the strong association between biofilm formation and antibiotic resistance reported in the current and prior studies (Madduri et al., 2025). In the context of MDR *K. pneumoniae*, biofilm formation may be particularly robust, which was observed in our finding where isolates resistant to ≥5 antibiotics included 66.7% strong biofilm producers.

The antibiofilm properties of ODHP-NS represent a strategically valuable activity. Checkerboard assay analysis revealed predominantly synergistic to additive interactions between ODHP-NS and ceftriaxone (CRO) against MDR *K. pneumoniae* isolates, with FICI values ≤ 0.5 at most of the tested concentration combinations, and no antagonistic interactions were detected. This is a noteworthy finding given that ceftriaxone exhibited MIC_50_ and MIC_90_ values of 512 µg/mL against the tested isolates indicating near-universal resistance among them. The ability of ODHP-NS to restore or significantly enhance the activity of ceftriaxone against these highly resistant strains suggests pharmacological synergy which can be applied clinically. The FICI-based synergy observed in the present study is consistent with findings from other investigations of unconventional antimicrobials combined with β-lactam antibiotics against resistant *K. pneumoniae*. For example, a 2024 study evaluating third-generation cephalosporins combined with polymyxin B against carbapenem-polymyxin-resistant *K. pneumoniae* reported FICI values of ≤ 0.5 for nearly all tested strain-combination pairs, with >4-fold reductions in required antibiotic dosages (Sturaro et al., 2024). Similarly, synergistic interactions between ceftazidime-avibactam and fosfomycin, colistin, or aminoglycosides have been documented against carbapenem-resistant *K. pneumoniae* (CRKP), underscoring that combination approaches can meaningfully extend the therapeutic utility of conventional antibiotics against resistant pathogens (Tüzemen et al., 2024).

The mechanistic basis for synergy between ODHP-NS and ceftriaxone likely involves the ability of ODHP (similar to other phenolic compounds) to disrupt bacterial membrane integrity or inhibit efflux pump activity, thereby facilitating the accumulation of ceftriaxone at the penicillin-binding proteins involved in cell-wall synthesis (Peng et al., 2025). Phenolic esters with long-chain acyl groups, such as ODHP, are known to interact with lipid bilayers, increasing membrane permeability (Durand et al., 2017). Increased permeability would enhance the intracellular concentration of ceftriaxone, even in strains expressing resistance mechanisms based on reduced outer membrane permeability (OmpK35/OmpK36 loss) (Rocker et al., 2020) or efflux (Duda-Madej et al., 2025). This hypothesis is supported by the observation that synergy was most pronounced at concentrations of ODHP-NS (32–64 µg/mL) that are above its 1/4 MIC for most isolates, suggesting a membrane-active effect on top of direct antibacterial activity. It is important to note that the proposed mechanisms (membrane disruption and efflux pump inhibition) are hypotheses based on the known activities of phenolic compounds (as suggested by studies such as Peng et al. (2025) and the observed synergy. Direct experimental validation (e.g., membrane permeability assays using fluorescent probes, efflux pump activity measurements using ethidium bromide accumulation) was beyond the scope of the present study and will be addressed in future investigations.

Time–kill kinetic analysis confirmed the checkerboard findings, demonstrating a markedly enhanced bactericidal effect of the ODHP-NS/CRO combination compared to either agent alone. The combination achieved a ≥3 log_10_ reduction in bacterial counts by 24 h, compared to the most active single agent, with no evidence of bacterial regrowth throughout the incubation period. This bactericidal threshold is particularly significant, as a ≥2 log_10_ reduction compared to the most active single agent is the conventional criterion for defining synergy in time–kill assays (Zhou et al., 2020). While the checkerboard assay successfully provided a static, quantitative index (FICI) of drug interaction, it is an endpoint measurement that cannot capture temporal killing effects. Therefore, time-kill assays were essential to validate the true bactericidal nature of the synergy and to confirm that the ODHP-NS and ceftriaxone combination prevents bacterial regrowth over time. The sustained bactericidal activity without regrowth suggests that the combination effectively overwhelms the adaptive responses and resistance mechanisms of MDR *K. pneumoniae*, including potential β-lactamase induction and efflux pump upregulation that may occur in response to single-agent treatment.

The rapid onset of bactericidal activity (significant reduction by 4 h) observed with the ODHP-NS/CRO combination is clinically relevant. Early bactericidal action can reduce the bacterial burden before adaptive resistance mechanisms are induced, limiting the selection of resistant mutants. This is in contrast to ceftriaxone alone, which showed limited activity consistent with the high MIC values observed, and ODHP-NS alone, which suppressed but did not eliminate the bacterial population over 24 h. The combination thus demonstrates a pharmacodynamic complementarity that neither agent achieves independently, providing a rationale for its further development. The results of this study support growing evidence that nanosponge-based drug delivery systems represent a promising platform for enhancing the antimicrobial activity of naturally derived and semisynthetic compounds. Nanosponges offer several inherent advantages relevant to antimicrobial applications: three-dimensional porous architectures that enable high drug loading, biocompatibility, controlled and sustained drug release, protection of encapsulated agents from enzymatic degradation, and the capacity to encapsulate both hydrophilic and hydrophobic molecules (Koppula and Maddi, 2024). These properties collectively allow nanosponge formulations to overcome key pharmacokinetic limitations of hydrophobic natural compounds, such as poor aqueous solubility, limited tissue penetration, and rapid systemic clearance. In the context of infectious disease, nanosponge technology has been explored for the delivery of various antimicrobial agents, with demonstrated improvements in solubility, bioavailability, and antibacterial potency (Koppula and Maddi, 2024). The present study adds to this evidence base by demonstrating that ODHP-NS achieves significantly lower MIC values than the free compound against a clinically relevant panel of MDR *K. pneumoniae* isolates, with no isolates requiring concentrations ≥256 µg/mL. The manufacturing process for ODHP-NS, based on the emulsion solvent diffusion method previously described (El-Sayed et al., 2024), is scalable and reproducible, supporting the translational potential of this formulation approach.

The nanotechnology-based approach to combating MDR *K. pneumoniae* is increasingly recognized as a strategic priority. Polymeric nanoparticle systems, including nanosponges, metal oxide nanoparticles, and lipid nanoparticles, have been shown to overcome multiple resistance mechanisms simultaneously through broad-spectrum membrane disruption, reactive oxygen species generation, and bypassing of efflux pump-mediated resistance (Hayat et al., 2025). The dual antibacterial and antibiofilm activity of ODHP-NS demonstrated in the present study, combined with its capacity to synergize with conventional antibiotics, positions it as a particularly versatile candidate within this emerging therapeutic category.

The present study offers several notable strengths. The evaluation was conducted against a clinically representative collection of 28 MDR *K. pneumoniae* isolates, providing ecological validity to the findings. Comprehensive methodological approaches were employed, including MIC determination by broth microdilution, quantitative biofilm inhibition assays, checkerboard synergy analysis, and confirmatory time–kill kinetics, providing a multi-dimensional characterization of the antimicrobial profile of ODHP-NS. Statistical analyses were performed using rigorous non-parametric methods appropriate for the distribution of microbiological data. However, several limitations should be acknowledged. The study is limited to *in vitro* experimentation; the *in vivo* pharmacokinetics, bioavailability, toxicity, and efficacy of ODHP-NS remain to be established in animal infection models. The mechanisms underlying the antibacterial and antibiofilm activities of ODHP and the mechanistic basis of its synergy with ceftriaxone have not been characterized at the molecular level in the present study, and future investigations employing membrane permeability assays, efflux pump activity measurements, and proteomics will be important for elucidating these mechanisms. Additionally, the molecular basis of multidrug resistance in the tested isolates, including specific β-lactamase genes (e.g., *bla*_KPC_, *bla*_NDM_, *bla*_OXA-48_) and resistance-associated mutations, was not characterized in the present study. The present study also lacks a direct comparison with other nanosponge-encapsulated natural products or alternative nanocarriers. Future studies should include such comparisons to contextualize the fold-improvement observed with ODHP-NS relative to other systems, and should prioritize: (i) *in vivo* evaluation of ODHP-NS pharmacokinetics and efficacy in murine and *Galleria mellonella* infection models; (ii) molecular characterization of resistance determinants and their correlation with ODHP-NS susceptibility; (iii) mechanistic studies of membrane disruption, efflux inhibition, and biofilm matrix degradation by ODHP-NS; (iv) cytotoxicity and biocompatibility assessment of ODHP-NS in mammalian cell lines; and (v) exploration of the ODHP-NS/ceftriaxone combination in *in vivo* infection models. Such studies would provide the translational evidence required to advance ODHP-NS toward clinical evaluation as an antimicrobial adjuvant for MDR *K. pneumoniae* infections.

## 5 Conclusion

This study demonstrates that nanosponge encapsulation of ODHP significantly enhances its antibacterial and antibiofilm activities against MDR *K. pneumoniae* and effectively potentiates the activity of ceftriaxone in a synergistic combination. ODHP-NS exhibited lower MIC values, greater inhibition of biofilm formation at sub-inhibitory concentrations, and pronounced bactericidal synergy in combination with ceftriaxone, as confirmed by time–kill kinetics. These findings establish ODHP-NS as a promising antimicrobial adjuvant with multi-modal activity against a high-priority MDR pathogen, highlighting the need for further preclinical and translational investigation.

## Data Availability

The original contributions presented in the study are included in the article/**Supplementary Material**. Further inquiries can be directed to the corresponding author.
