# Supplementary material for: Octadecyl 3-(3, 5-di-tert-butyl-4-hydroxyphenyl) propanoate nanosponges: enhanced antibacterial and antibiofilm activity against multidrug-resistant Klebsiella pneumoniae with synergistic ceftriaxone combination
Source: Front Cell Infect Microbiol. 2026 Jul 16;16:1867536. doi: 10.3389/fcimb.2026.1867536 (PMC13422199; doi:10.3389/fcimb.2026.1867536)
Supplement: Supplementary file 1 [file DataSheet1.docx]

**Supplementary Material**

**ORIGINAL RESEARCH**


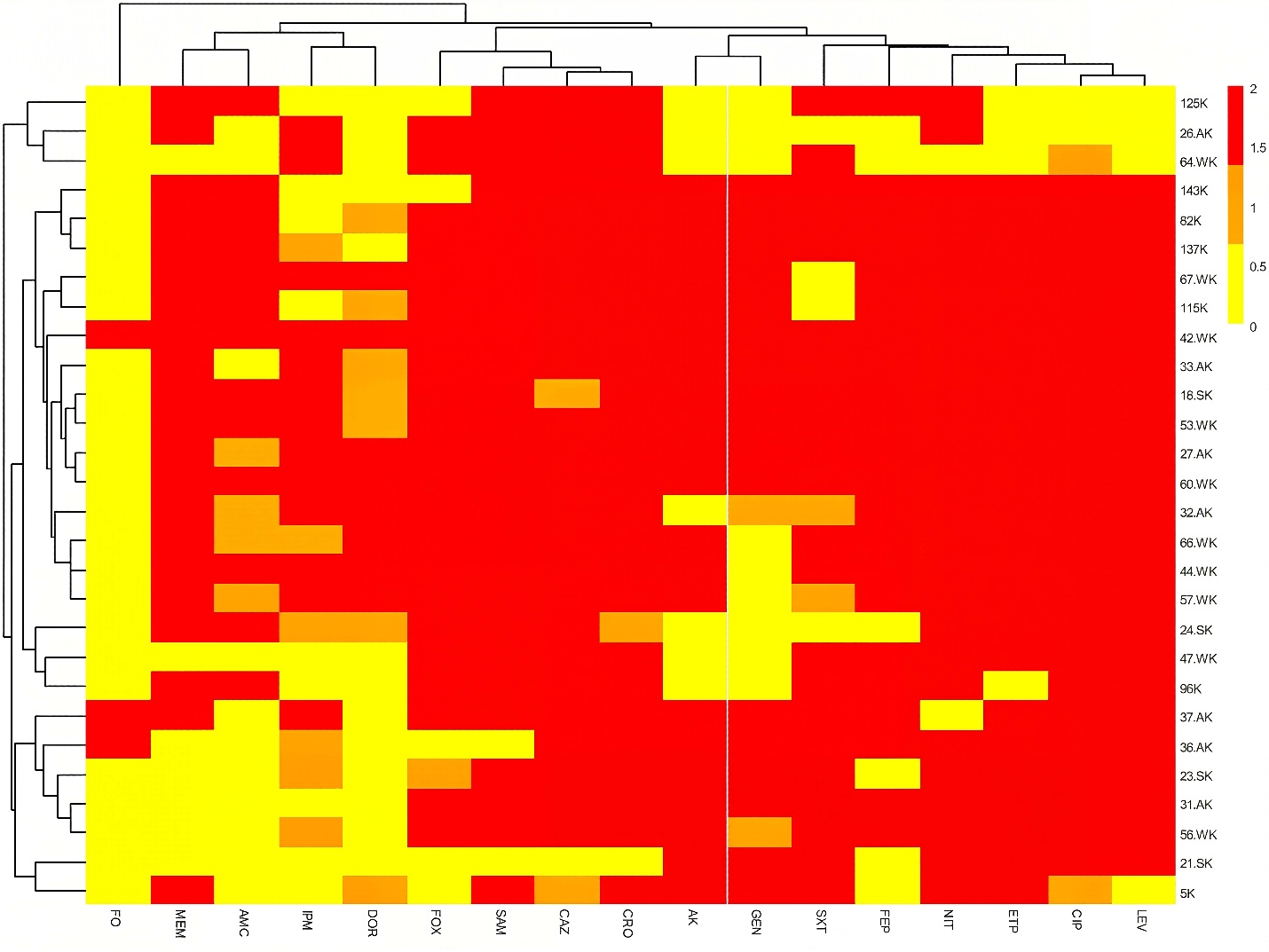
**Octadecyl 3-(3,5-di-tert-butyl-4-hydroxyphenyl) propanoate Nanosponges: Enhanced Antibacterial and Antibiofilm Activity Against Multidrug-Resistant *Klebsiella pneumoniae* with Synergistic Ceftriaxone Combination**

**Supplementary Figure S1.** Hierarchical clustering heatmap illustrating the antimicrobial susceptibility patterns of *Klebsiella pneumoniae* isolates. Each row represents an individual isolate, and each column corresponds to a tested antibiotic. The color gradient reflects susceptibility status (red = resistant, yellow = intermediate, lighter shades = susceptible). Dendrograms indicate similarity relationships among isolates and antibiotics based on their resistance profiles, highlighting clustering of multidrug-resistant phenotypes and shared resistance patterns across antimicrobial classes.
